# Successful Conservative Management of Traumatic Pancreatic Duct Injury: A Case Report

**DOI:** 10.7759/cureus.27749

**Published:** 2022-08-07

**Authors:** Mohammed Abdullah, Khalid Babieker, Ali A Almohammed Saleh

**Affiliations:** 1 General Surgery, King Fahad General Hospital, Al-Ahsa, SAU; 2 Medical Intern, King Faisal University, Al-Ahsa, SAU

**Keywords:** pancreatic injury, pancreatectomy, conservative management, pancreatic duct injury, blunt abdominal injury

## Abstract

Patients with blunt abdominal trauma are at high risk of getting pancreatic injury, with high morbidity and mortality rates. The decision to manage the patient operatively depends highly on the condition of the main pancreatic duct (MPD). Conservative management of traumatic MPD injuries is rarely reported in the literature. In this article, we present the case of a 19-year-old male patient, medically free, admitted to the hospital following blunt abdominal trauma. He was hemodynamically stable and computed tomography showed MPD injury with no evidence of active bleeding or bile leakage. So, the decision to manage him conservatively was taken. Our aim in this report is to highlight the fact that the operative management of traumatic MPD injuries, which carries dangerous post-operative complications, could be avoided.

## Introduction

Traumatic pancreatic injury is uncommon and hard to diagnose, mostly because of the retroperitoneal location of the pancreas [1]. Patients with blunt abdominal trauma are highly susceptible to getting a pancreatic injury, with a 30% mortality and morbidity reaching up to 45% [2]. The way of managing patients with traumatic pancreatic injury depends highly upon the state of the main pancreatic duct (MPD). Non-operative management of minor traumatic pancreatic injuries is widely accepted. However, traumatic disruption of the MPD has been known to be an indication of exploratory laparotomy [3]. We hereby present a case report of a 19-year-old male patient who has a history of blunt trauma one month ago and presented to the emergency department with abdominal pain and vomiting. He was diagnosed with a case of pancreatic pseudocyst with MPD injury. The patient was managed conservatively and discharged home on day 20 post-admission.

## Case presentation

**Figure 1:**
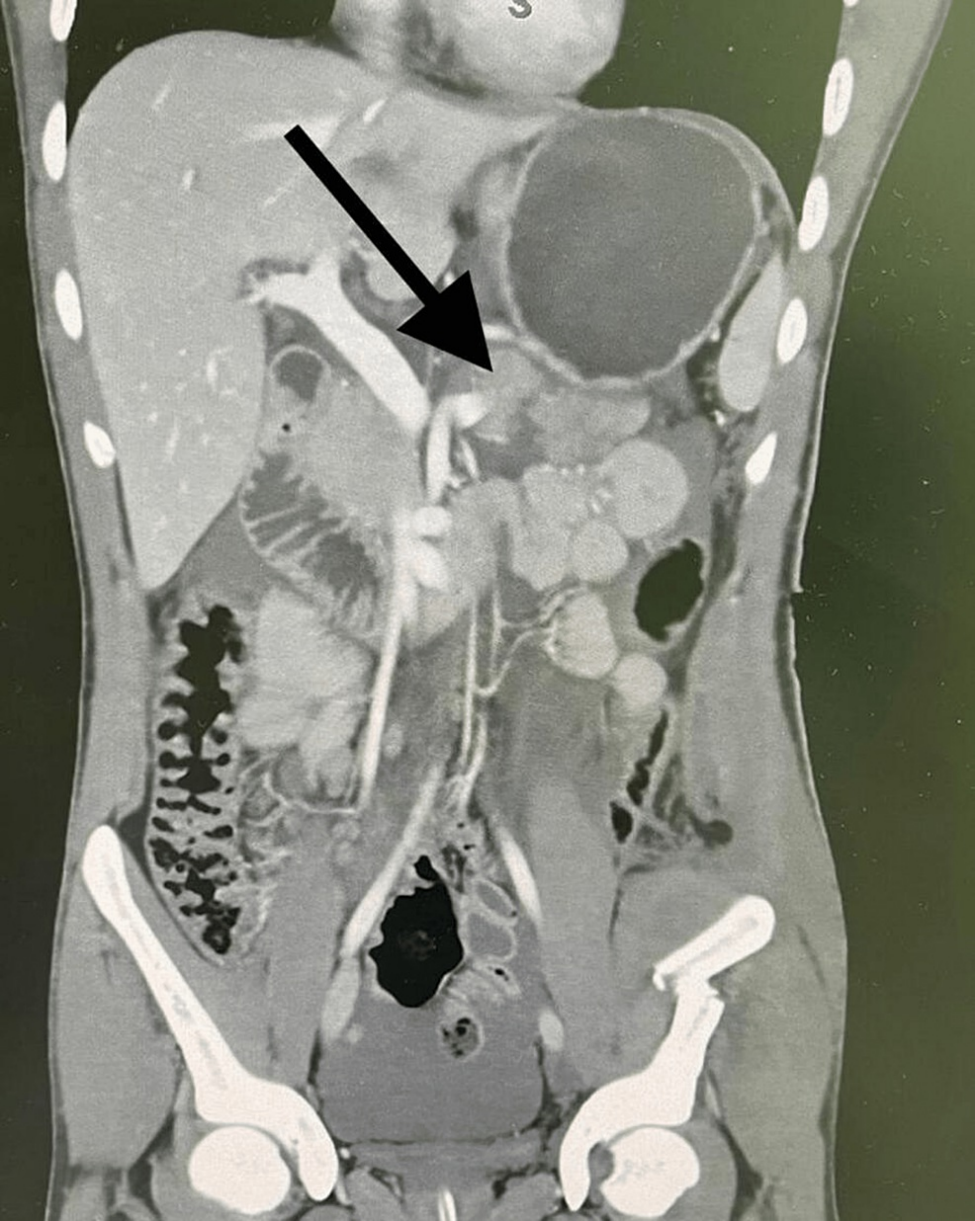
CT scan with contrast of the abdomen and pelvis, coronal view showed laceration through the body of the pancreas and peripancreatic fluid collection.

A 19-year-old male patient, medically free, admitted to the hospital immediately after blunt abdominal trauma. He was vitally stable, and the physical examination showed a conscious, alert, and oriented patient. An abdominal examination revealed epigastric tenderness and guarding. Laboratory investigations showed the following: white blood cells (WBC) 13.2 × 10^9^/L (normal range is 4-10 × 10^9^/L), hemoglobin 11.7 g/dL (normal range is 13-17 g/dL), normal level of total and direct bilirubin, and serum amylase 607 U/L (normal range is 20-115 U/L). An urgent computed tomogram (CT) was performed which showed a laceration through the body of the pancreas that extends from its anterior to posterior borders, denoting pancreatic transection. Furthermore, there was a peripancreatic fluid collection in the lesser sac of the pancreas that resulted in anterior displacement of the stomach, which was highly suggestive of pancreatic duct injury. There was no evidence of active bleeding or bile leakage (Figure 1).

**Figure 2:**
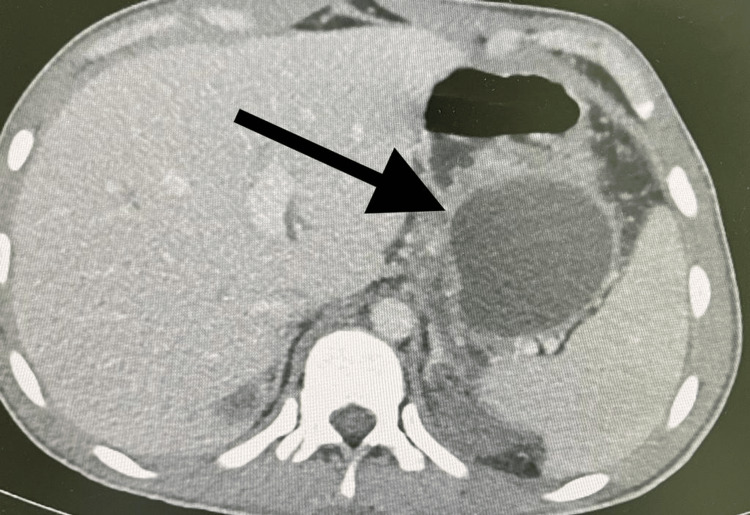
CT scan with contrast of the abdomen and pelvis, axial view showing pancreatic pseudocyst.

So, conservative management for the patient was decided. He was kept nil by mouth with intravenous hydration and intravenous antibiotics. Since the patient was in a stable condition and started to improve clinically, a low-fat diet was introduced, and intravenous antibiotics were stopped on the seventh day of admission. A further CT scan was performed on day 17 post-admission which revealed a stable appearance of pancreatic body laceration with a well-defined cystic lesion which was observed at the tail of the pancreas measuring about 6 × 6.5 × 7, denoting pancreatic pseudocyst (Figure 2).

The patient remained hemodynamically stable and gradually improved both clinically and biochemically, so he was discharged on day 20. He continues to be monitored in the outpatient setting and remains clinically well with normal pancreatic endocrine and exocrine functions as of five years since discharge.

## Discussion

The pancreas is a gland that is located in the retroperitoneal position. So, pancreatic injury from blunt abdominal trauma is relatively infrequent [4]. It occurs in only 3% to 12% of all patients with severe abdominal trauma [5]. Furthermore, many pancreatic injuries are asymptomatic and difficult to detect. They become apparent when complications emerge or other abdominal organ injuries take place. More than 80% of patients with pancreatic injuries have at least one other abdominal organ that is also injured [6]. Pancreatic injury is associated with high morbidity and mortality rates [7,8]. However, there are no signs, symptoms, or even laboratory findings that are specific to pancreatic injury, which leads to delayed diagnosis and significant complications like pseudo-cyst, abscess formation, and sepsis [9]. A CT scan is considered a simple, non-invasive method for the initial assessment of traumatic pancreatic injury [10]. The determining factor in the management of pancreatic injury is the condition of the MPD. When a large hematoma or deep laceration of the pancreatic parenchyma is seen on CT, MPD injury should be suspected and the patient should be managed accordingly [11]. Magnetic resonance cholangiopancreatography (MRCP) is another non-invasive method that allows the detection of MPD injury in hemodynamically stable patients. However, it does not enable the confirmation of ductal communication with a pancreatic pseudocyst or other fluid collection [12].

Pancreatic trauma is classified according to the pancreatic organ injury scale of the American Association for the Surgery of Trauma (AAST). The MPD is preserved in grades I and II, and therefore patients in this category are managed conservatively [13]. When there is distal transection or parenchymal injury with duct injury, this is called grade III. Distal pancreatectomy has been known to be the definitive management of pancreatic injuries where there is ductal injury [14]. However, inadequate resection may lead to many post-operative complications, which include bleeding, pancreatic fistula, abscess, pseudocyst, and persistent pancreatic pain [10]. The mortality rate of surgically managed pancreatic injuries reaches up to 34.8%, while the morbidity rate has been shown to be 52.2% [15]. Pancreatic duct stent placement has been shown to be an effective method of managing pancreatic MPD injury [11,16,17]. However, ductal stricture at the injured site is a major long-term complication of this procedure [18].

We presented a case of a patient with an MPD injury that has been managed conservatively with good outcomes. The dangerous post-operative complications of traumatic pancreatic duct injury have been avoided with successful conservative management. Our patient was hemodynamically stable with no evidence of active hemorrhage or bile leak on imaging, so the decision to treat him conservatively was taken.

## Conclusions

Conservative management appears to be applicable in selected patients with traumatic MPD injury following abdominal trauma. This allows the patient to avoid potentially dangerous post-operative complications, which carry a high risk of morbidity and mortality. The way of treating the patient with traumatic MPD injury should be according to the AAST classification as well as the patient's clinical status.
